# Optimizing colorectal polyp detection and localization: Impact of RGB color adjustment on CNN performance

**DOI:** 10.1016/j.mex.2025.103187

**Published:** 2025-01-27

**Authors:** Jirakorn Jamrasnarodom, Pharuj Rajborirug, Pises Pisespongsa, Kitsuchart Pasupa

**Affiliations:** a Faculty of Medicine, King Mongkut's Institute of Technology Ladkrabang, Bangkok, Thailand; b School of Information Technology, King Mongkut's Institute of Technology Ladkrabang, Bangkok, Thailand

**Keywords:** Colonoscopy, Colonic polyp, Computer-assisted image analysis, Artificial intelligence, Image preprocessing, Bayesian-Optimized RGB Color Adjustment for CNN Performance Enhancement

## Abstract

Colorectal cancer, arising from adenomatous polyps, is a leading cause of cancer-related mortality, making early detection and removal crucial for preventing cancer progression. Machine learning is increasingly used to enhance polyp detection during colonoscopy, the gold standard for colorectal cancer screening, despite its operator-dependent miss rates. This study explores the impact of RGB color adjustment on Convolutional Neural Network (CNN) models for improving polyp detection and localization in colonoscopic images. Using datasets from Harvard Dataverse for training and internal validation, and LDPolypVideo-Benchmark for external validation, RGB color adjustments were applied, and YOLOv8s was used to develop models. Bayesian optimization identified the best RGB adjustments, with performance assessed using mean average precision (mAP) and F_1_-scores. Results showed that RGB adjustment with 1.0 R-1.0 G-0.8 B improved polyp detection, achieving an mAP of 0.777 and an F_1_-score of 0.720 on internal test sets, and localization performance with an F_1_-score of 0.883 on adjusted images. External validation showed improvement but with a lower F_1_-score of 0.556. While RGB adjustments improved performance in our study, their generalizability to diverse datasets and clinical settings has yet to be validated. Thus, although RGB color adjustment enhances CNN model performance for detecting and localizing colorectal polyps, further research is needed to verify these improvements across diverse datasets and clinical settings.

• **RGB Color Adjustment**: Applied RGB color adjustments to colonoscopic images to enhance the performance of Convolutional Neural Network (CNN) models.
• **Model Development**: Used YOLOv8s for polyp detection and localization, with Bayesian optimization to identify the best RGB adjustments.
• **Performance Evaluation**: Assessed model performance using mAP and F_1_-scores on both internal and external validation datasets.

## Graphical abstract

**Figure:**
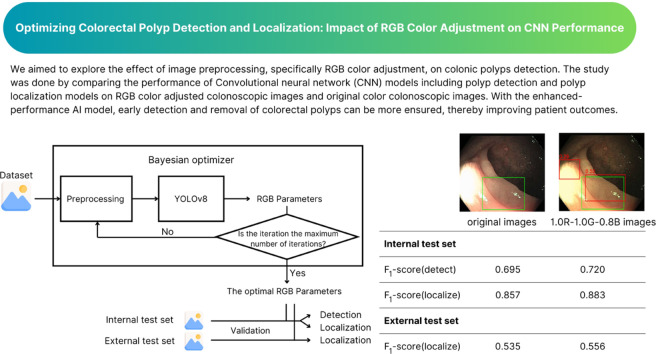

Specifications table

**Table:** 

Subject area:	Bioinformatics
More specific subject area:	Image preprocessing
Name of your method:	Bayesian-Optimized RGB Color Adjustment for CNN Performance Enhancement
Name and reference of original method:	- Reis D KJ, Hong J, et al. Real-time flying object detection with yolov8: arXiv; 2023 [2024 Feb 5]. Available from: https://arxiv.org/abs/2305.09972. - Zhang Y, Sohn K, Villegas R, Pan G, Lee H, editors. Improving object detection with deep convolutional networks via Bayesian optimization and structured prediction. 2015 IEEE Conference on Computer Vision and Pattern Recognition (CVPR); 2015 7–12 June 2015. - S.Gopal Krishna Patro KKs. Normalization: A Preprocessing Stage. International Advanced Research Journal in Science, Engineering and Technology 2015;2(3). - S R. Model evaluation, model selection, and algorithm selection in machine learning arXiv; 2018 [updated 2020 Nov 112,024 Feb 5]. Available from: https://arxiv.org/abs/1811.12808.
Resource availability:	Dataset - Wang G. Replication Data for: Colonoscopy Polyp Detection and Classification: Dataset Creation and Comparative Evaluations. V1 ed: Harvard Dataverse; 2021. - Ma Y, Chen X, Cheng K, Li Y, Sun B, editors. LDPolypVideo Benchmark: A Large-Scale Colonoscopy Video Dataset of Diverse Polyps. Medical Image Computing and Computer Assisted Intervention – MICCAI 2021; 2021 2021//; Cham: Springer International Publishing.

## Background

Colorectal cancer is the third most common cancer globally [1]. It ranks as the fourth leading cause of cancer-related death in Thailand and the second leading cause of cancer mortality worldwide [2]. According to the adenoma-carcinoma sequence theory, colorectal cancer often develops from non-cancerous polyps. However, if the adenomatous polyp is left untreated, it may develop into a malignant tumor that is life-threatening[3]. Therefore, the detection and removal of adenomatous polyps can play a significant role in preventing colorectal cancer [4].

Colonoscopy is one of the reference standards for detecting colorectal polyps and is the most sensitive method [5]. It is efficient in detecting colonic lesions and polyps. It also enables the removal of precancerous polyps and biopsy lesions by physicians. However, colonoscopy is an operator-dependent procedure, and polyp miss rate as high as 21–28 % has been reported in certain cases[[6], [7], [8]].

One of the techniques that help physicians enhance their colonoscopic performance is Image-enhanced Endoscopy (IEE), such as Narrow Band Imaging (NBI), which uses optical filters to enhance features of polyps, thereby maximizing the detection of colorectal polyps [9]. NBI eliminates red light using optical filters and narrows the bandwidth to a range where only blue and green light are visible. Green light penetrates and highlights deeper blood vessels, whereas blue light highlights the architecture of the surface capillaries.

A number of artificial intelligence techniques, particularly Convolutional Neural Networks (CNNs), have been developed to assist clinicians in detecting polyps and tumors during colonoscopy. These techniques help reduce the polyp miss-rate and optimize the screening process. CNNs are a type of artificial intelligence designed to process and analyze image data by using convolutional layers to detect patterns and features. Various computer vision techniques, which involve using artificial intelligence to enable computers to extract meaningful data from visual inputs are utilized. These techniques include object recognition techniques, image classification, image segmentation, and object detection. Based on the foundation that features extraction is the backbone of computer vision, using preprocessing, which is similar to IEE, might allow the CNN model to extract more features and improve performance. However, there was a limited number of studies on artificial intelligence for colorectal polyp detection and image preprocessing. Chen-Ming Hsu et al. utilized grayscale images and the Faster Regional Convolutional Neural Network (Faster R-CNN) architecture, one of famous object detection models for polyp detection. Their results showed that the mean accuracy for polyp detection of grayscale images was approximately 1 % higher than that of RGB/NBI images [10]. Zhiqin Qian et al. also conducted a study, which preprocessed colonoscopic images in HSV color space to adjust specular reflection before subsequently analyzed with the tailored Faster R-CNN algorithm. The mAP achieved by their approach was 91.43 %, surpassing the 90.57 % attained by the Faster R-CNN alone [11].

Therefore, we aimed to explore the effect of image preprocessing, specifically RGB color adjustment, on polyp detection CNN model. RGB color adjustment involves modifying the intensity of red, green, and blue channels in an image to enhance specific features. This technique can improve the visibility of polyps in colonoscopic images, making it more effective than traditional methods, such as grayscale conversion. Other color adjustment techniques, such as HSV color space adjustment and grayscale conversion, have been explored in previous studies. We chose to study RGB color adjustment because the NBI optical filter, which enhances the features of polyps during colonoscopy, is also based on the RGB adjustment principle. Similar to NBI, RGB adjustment enhances the visibility of polyps, potentially reducing the polyp miss rate and improving early detection of colorectal cancer.

## Method details

This study was a cross-sectional study that compared the performance of colorectal polyp detection and localization models (CNN models) on RGB adjusted colonoscopic images with the performance on original color colonoscopic images. The aim was to visualize the effect of RGB color adjustment on CNN model for polyp detection and polyp localization. The polyp detection CNN model used in this study took an image as input specifically pixels in the image and produced an output as bounding boxes and the probability of predicted classes for the detected polyps, which were adenoma and hyperplastic polyp. Each pixel in the image represented red, green, and blue pixels. The adjustment was applied to the model's input by multiplying RGB hyperparameters with the red, green and blue pixel values of the input image.

### Dataset

The Harvard dataverse, which combines four datasets including – MICCAI 2017, CVC colon DB, GLRC dataset, and KUMC dataset – was selected for the training, internal validation, and internal test set. The choice was made because the dataset included both bounding boxes and annotations, allowing us to perform both detection and localization tasks, which were the primary objective of this study. Additionally, the dataset's generalizability was enhanced by combining multiple datasets. The dataset was divided into training, validating, and test sets containing 28,773, 4254, and 4872 images, respectively, and included two categories of polyps: hyperplastic and adenomatous polyps [12,13]. Each set was used as its designation in our study.

The LDPolypVideo-Banchmark was chosen as the external test set because it contained only bounding boxes, which unfortunately limited its use to detection tasks. The dataset included 40,266 frames of colonoscopic snapshot [14].

### Algorithm

#### YOLOv8s

The YOLOv8s algorithm was used to develop a polyp detection and localization model. YOLO (You Only Look Once) is a real-time object detection algorithm that divides an image into a grid and predicts bounding boxes and class probabilities for objects in a single evaluation [15]. YOLOv8 stands as the most recent iteration of the YOLO object detection model, maintaining the same underlying architecture as its predecessors. However, it brings forth several enhancements when compared to earlier YOLO versions [16]. Notably, it introduces a novel neural network structure incorporating both Feature Pyramid Network (FPN) and Path Aggregation Network (PAN) [17]. The FPN functions by systematically reducing the spatial resolution of the input image while concurrently increasing the number of feature channels. This process generates feature maps capable of recognizing objects at varied scales and resolutions. In contrast, the PAN architecture consolidates features from different network levels through skip connections, enabling the network to effectively capture features across multiple scales and resolutions. There are five models of YOLOv8s algorithm including n, s, m, 8l, and x models. The s model of YOLOv8 was selected because the model needed to be fast and have a small computational burden in order to work in real-time applications. We opted to use the unmodified YOLOv8s in this study since our study aim was not to develop the model but to visualize the effect of RGB adjustment.

#### Bayesian optimizer

To determine the effect of RGB adjustment, the effects of every combination of adjusted R, G, and B layers should be considered. However, it would cause a significant computational burden and processing time. Therefore, we employed a Bayesian optimizer, which is a method commonly used for hyperparameters tuning in machine learning, to find the best RGB combination for polyp detection task based on mAP of the YOLOv8s model. Bayesian optimization is a strategy for optimizing functions by building a probabilistic model, which is usually a Gaussian process, of the objective function and using it to select the most promising points to evaluate next, balancing exploration and exploitation. As the number of observations increases, the posterior distribution refines, enhancing the algorithm's certainty about which regions in the parameter space are worth exploring and which are not [18].

The hyperparameters, generated using the Bayesian optimizer, consisted of sets of weights for the adjustments of the R, G, and B layers, ranging from 0.0 to 2.0 with intervals of 0.1. This range was selected to cover a broad spectrum of possible adjustments, enabling the identification of the most effective combinations. The interval of 0.1 was selected as a balance between computational efficiency and granularity. A larger interval might overlook subtle yet important variations in performance, while a smaller interval would significantly increase the computational burden without necessarily providing proportionate benefits. The chosen interval ensures the capture of meaningful variations in performance without overwhelming computational resources.The rationale behind the specific hyperparameter sets chosen during Bayesian optimization is based on their ability to maximize the mAP of the YOLOv8s model. Bayesian optimization balances exploration and exploitation, ensuring that the selected hyperparameters are both effective and efficient. By exploring a wide range of hyperparameter values, the optimizer can identify combinations that yield the best performance. The probabilistic model used in Bayesian optimization helps to refine the search space, focusing on regions that are more likely to contain optimal solutions.

#### Preprocessing of colonoscopic images

Each set of weights for R, G, and B layers adjustment, which was generated in the Bayesian optimizer process, was used to preprocess the dataset before training the model. During preprocessing, all images’ colors were normalized using max-min normalization [19] to standardize the pixel values, and each image was then extracted into three color layers (red, green, and blue). The generated sets of weights were individually applied to each color layer as a multiplier and then merged back to form the processed images before use to train, validate, and test the models.

#### Cross-validation

After preprocessing, we employed five-fold cross-validation [20] on the RGB color adjusted and original color dataset with the YOLOv8s models. Cross-validation is a technique in machine learning used to evaluate a model's performance on unseen data. It involves splitting the data into multiple subsets, using one as a validation set while training the model on the others. This process is repeated with different validation sets, and the results are averaged to provide a more robust estimate of the model's performance.

### Experiment framework

The experimental framework was designed to evaluate the impact of RGB color adjustment on the performance of CNN models for colorectal polyp detection and localization using the YOLOv8s algorithm.

#### Finding a subset of training set for finding the optimal hyperparameter

Even though we planned to use the Bayesian optimizer to find the optimal hyperparameter, it would still cause a significant computational burden and processing time to train the model with the whole training set in every RGB color adjustment. Therefore, we tried to find the smallest subset of the training set that still made the model to have a similar performance as the whole training set could make. This was done by comparing the performance of the polyp detection model when training with randomly selected 10 %, 20 %, 40 %, 60 %, and 100 % of the original training set.

#### Finding the optimal hyperparameter and the effect of RGB adjustment on polyp detection

**Fig. 1:**
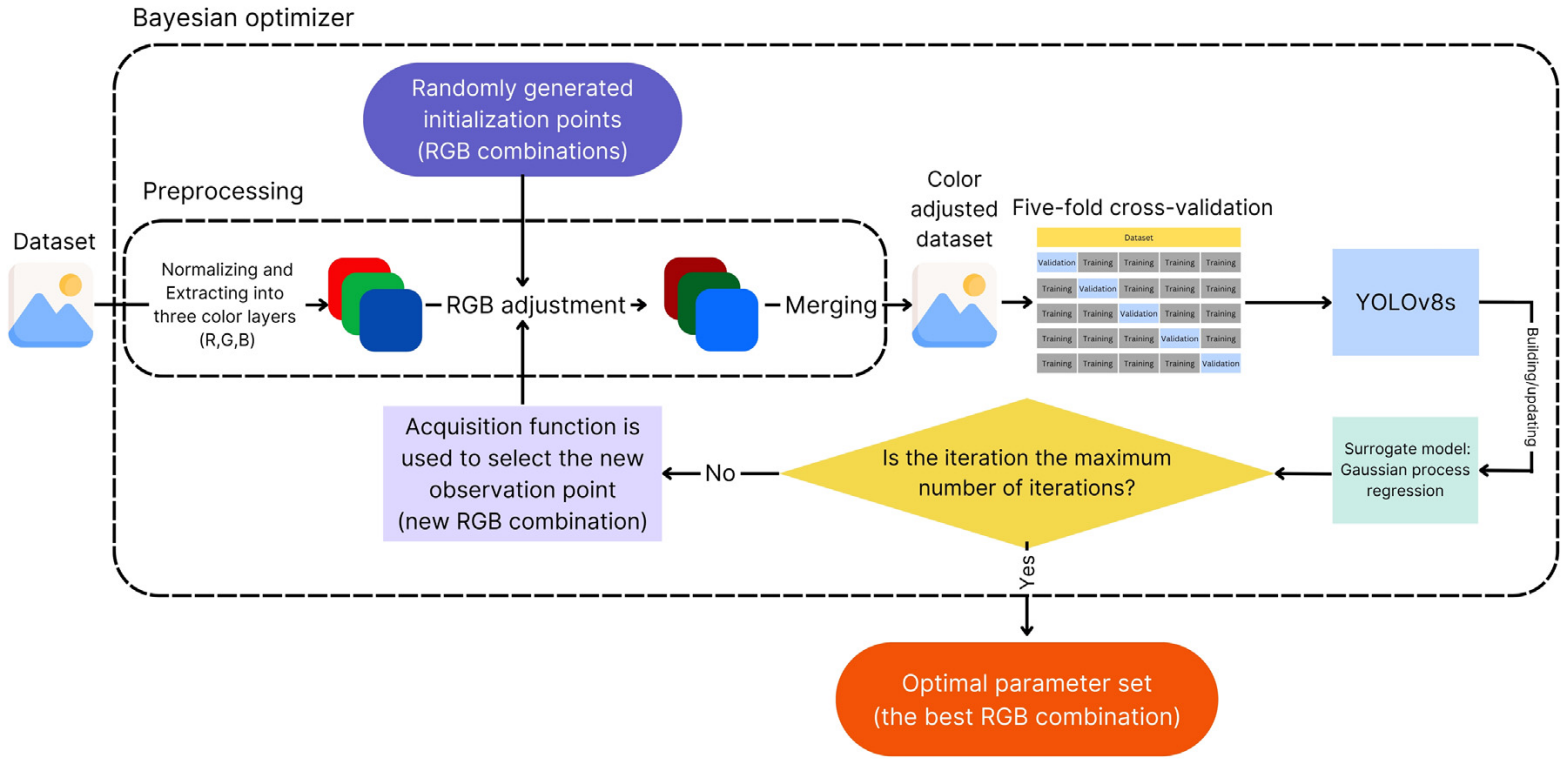
The flow representing the Bayesian optimizer with the process of preprocessing, five-fold cross-validation, and YOLOv8s model.

The Bayesian optimizer was utilized to identify the optimal RGB adjustment for polyp detection based on mAP. The settings of the Bayesian optimizer, which we used, were 10 random points for 100 iterations and 20 random points for 100 iterations. Randomly selected subset of training set and validation set were used in finding optimal RGB adjustment process. During data preprocessing, each image in the training and validation sets was normalized to standardized pixel values. Images were then extracted into red, green, and blue layers, adjusted according to the generated sets of weights by the Bayesian optimizer, and merged back to form processed images. In the model training and validation phase for each RGB combination, a five-fold cross-validation approach was employed to train and validate the YOLOv8s detection model, ensuring robust performance estimation (Fig. 1). At the end of the process, the two settings of the Bayesian optimizer provided the optimal RGB hyperparameters for each setting.

After obtaining these optimal RGB hyperparameters, we applied them to the entire dataset and trained the YOLOv8s polyp detection model accordingly. The trained model was then validated and tested using the validation and test sets, which were also adjusted with the same optimal RGB combinations. The performance of the polyp detection model on the two RGB adjusted colonoscopic images were compared with the performance of the model on the original dataset. Finally, the best hyperparameters from the two optimal hyperparameters were selected.

#### Finding the effect of RGB adjustment on polyp localization

The polyp localization model was trained, validated, and tested with the best RGB adjusted colonoscopic images. The performance of the polyp localization model on the best RGB adjusted colonoscopic images was compared with the performance of the model on the original dataset.

#### External validation

The trained polyp localization model was also tested with the best RGB adjusted external test set and original color external test set. The performances of the polyp localization on the RGB adjusted external set and the original external set were compared.

### Performance evaluation and statistical analysis

The performance of machine learning on polyp was assessed using precision, sensitivity, false positive, F_1_-score and mAP [20].

Precision indicates how many of the values estimated as positive are positive,

$$Precision=\;\frac{TP}{TP+FP},$$

where TP is true positive, and FP is false positive.

Recall indicates how many true positives are estimated from all positives. It is sometimes referred as sensitivity,

$$Sensitivity=\;\frac{TP}{TP+FN},$$

where FN is false negative.

The F_1_-score represents the harmonic mean of precision and recall metrics,

$$F_{1}=\;\frac{2\;\cdot Precision\cdot Recall}{Precision+Recall}.$$

mAP is a measure designed for evaluating the performance of models in object detection tasks and information retrieval on images [21]. It incorporates key metrics such as confusion matrix, Intersection over Union (IoU), Precision, and Recall,

$$mAP=\;\frac{\sum_{n=1}^{N}AveP\left(n\right)}{N},$$

where N is the number of classes in the set. AveP(n) is average precision of the class n.

The model's performance in polyp localization was reported as the polyp detection rate (sensitivity), precision, and F_1_-score. The analysis was conducted using Python version 3.9.7, developed by the Python Software Foundation in Delaware, US. A two-sided statistical significance level of 0.05 was chosen.

## Method validation

### Results

#### The best RGB combination and the overall performance of the polyp detection model on internal test set

The Bayesian optimizer resulted in the best hyperparameters of 1.1R-1.1G-1.0B and 1.0R-1.0G-0.8B from 10 random points for 100 iterations and 20 random points respectively. On the internal test set, the polyp detection model achieved a polyp detection mean average precision 50 (mAP_50_) of 0.761 on normal RGB images, 0.767 on modified 1.1R-1.1G-1.0B images, and 0.777 on modified 1.0R-1.0G-0.8B images. The F_1_-score of polyp detection was 0.695 on normal RGB images, 0.702 on modified 1.1R-1.1G-1.0B images, and 0.720 on modified 1.0R-1.0G-0.8B images (Table 2). Therefore, the 1.0 R-1.0 G-0.8 B combination was chosen as the better RGB combination.

#### Subgroup analysis of the performance of the polyp detection model on internal test set

In the subgroup analysis, we evaluated the performance of the polyp detection model based on the types of polyps: adenomatous and hyperplastic (Table 2). For adenomatous polyps, the model achieved mAP_50_ values of 0.671, 0.680, and 0.700 on the original color, 1.1R-1.1G-1.0B, and 1.0R-1.0B-0.8B images, respectively. The F_1_-scores were 0.587, 0.614, and 0.647 on the original color, 1.1R-1.1G-1.0B, and 1.0R-1.0B-0.8B images, respectively. For hyperplastic polyps, the model achieved mAP_50_ values of 0.851, 0.855, and 0.864 on the original color, 1.1R-1.1G-1.0B, and 1.0R-1.0B-0.8B images, respectively. The F_1_-scores were 0.790, 0.783, and 0.792 on the original color, 1.1R-1.1G-1.0B, and 1.0R-1.0B-0.8B images, respectively.

#### The performance of the polyp localization model on internal test set

**Fig. 2:**
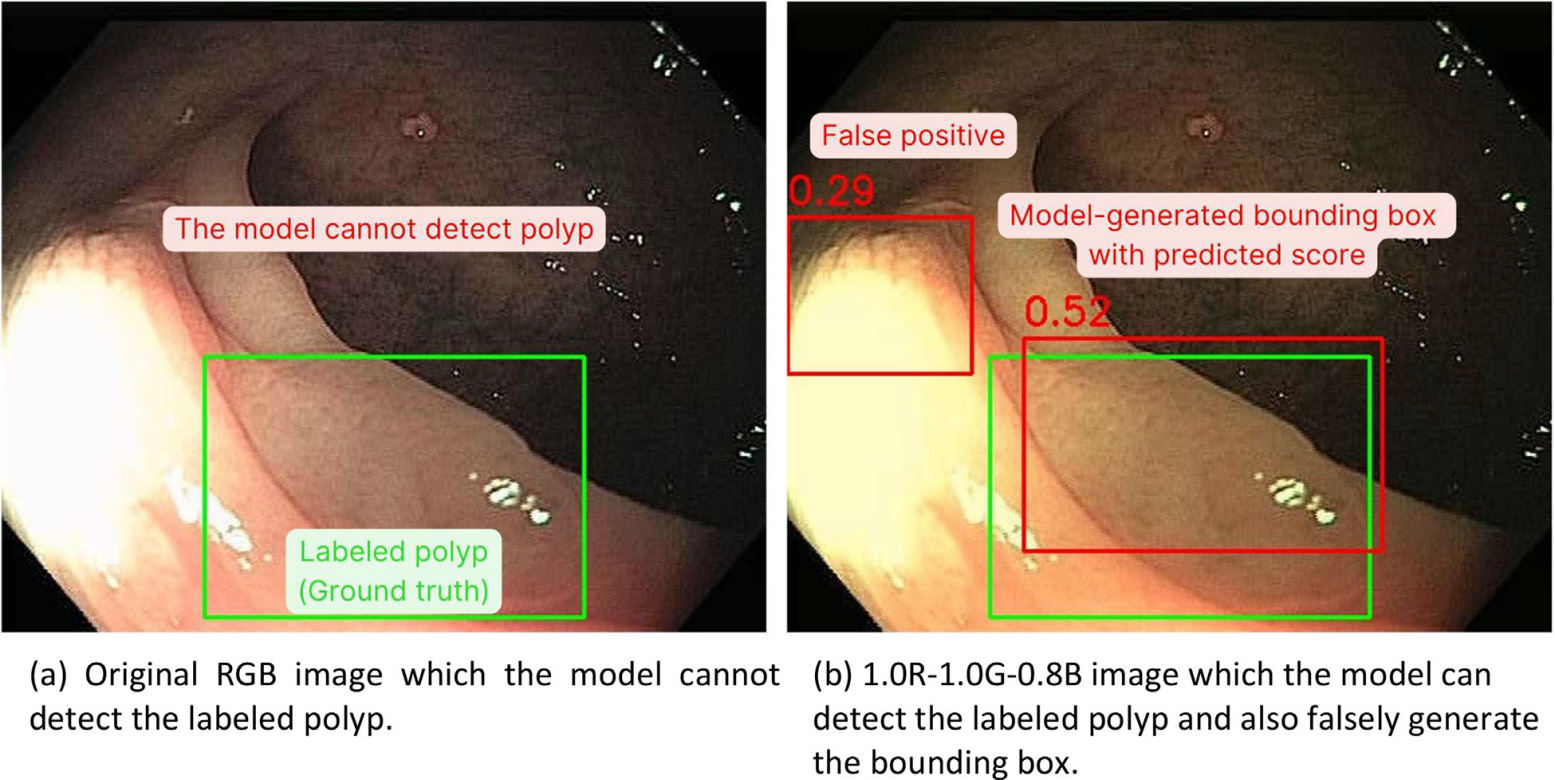
Enhanced polyp detection rate observed after image preprocessing, accompanied by an increase in false positives. (Green box: Ground truth of colorectal polyp in the image, Red box: Model-generated bounding box of colorectal polyp, Number: Predicted confidence score by the model).

**Fig. 3:**
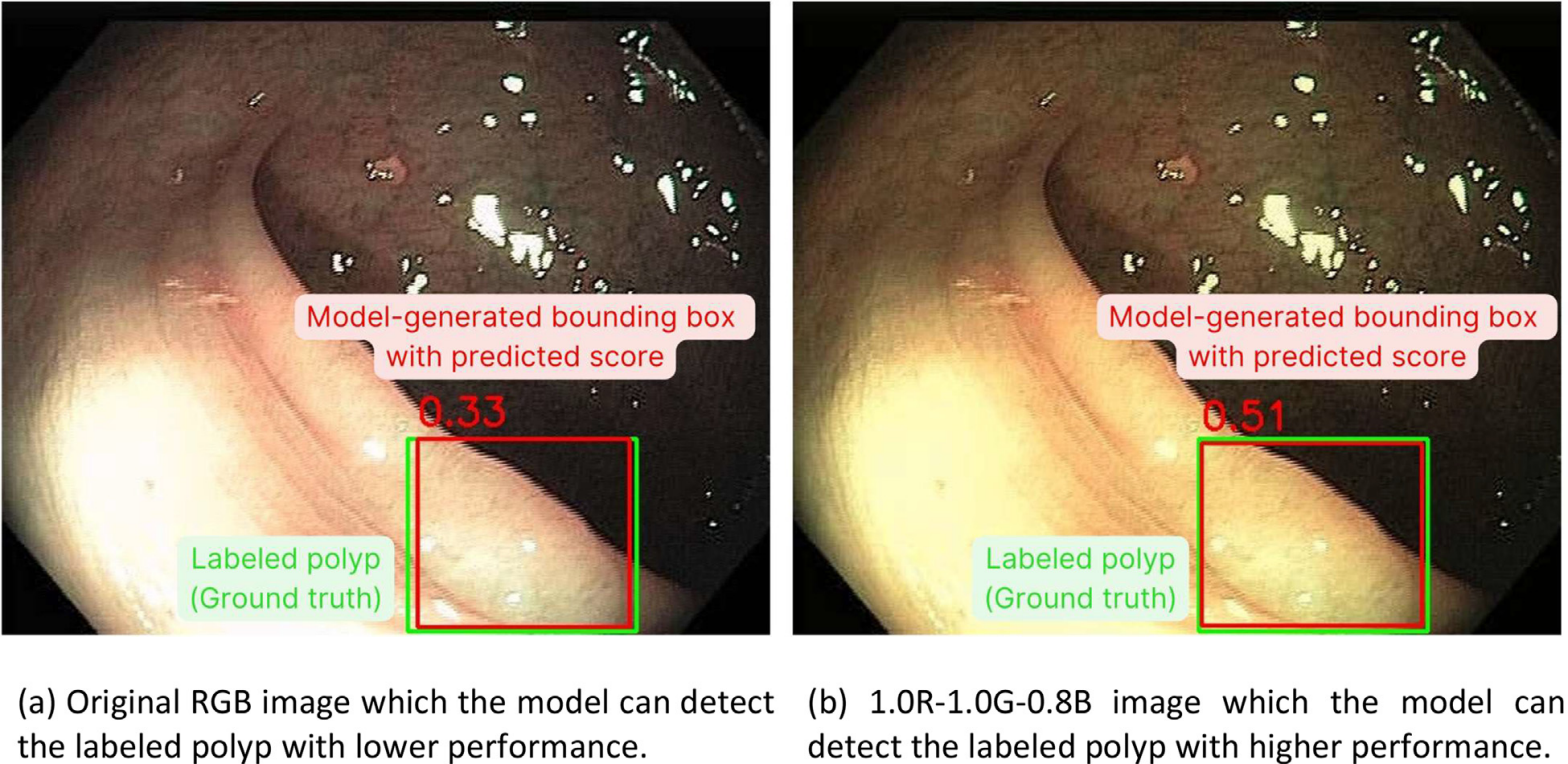
Enhanced polyp detection rate observed after image preprocessing. (Green box: Ground truth of colorectal polyp in the image, Red box: Model-generated bounding box of colorectal polyp, Number: Predicted confidence score by the model).

**Fig. 4:**
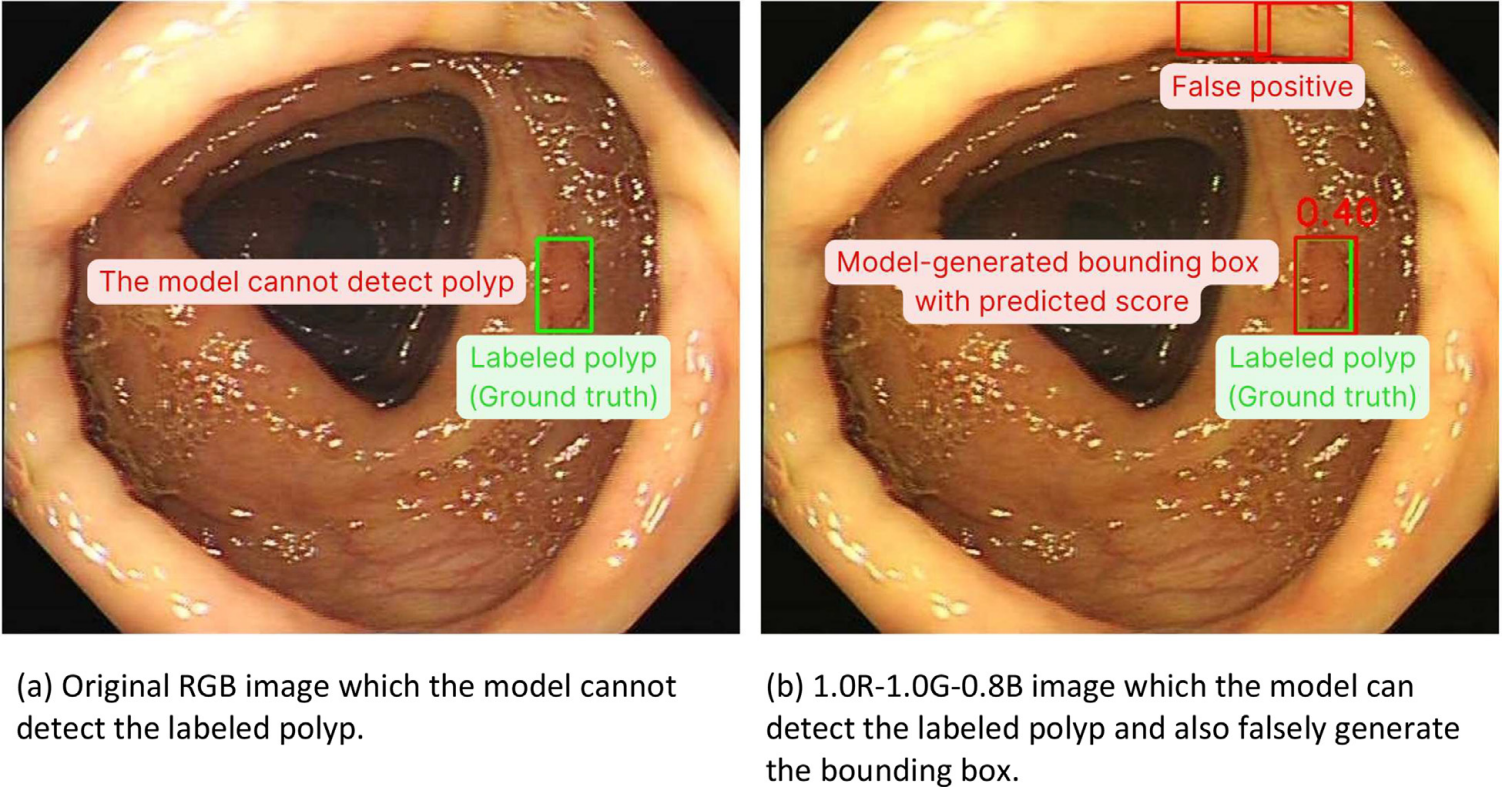
Enhanced polyp detection rate observed after image preprocessing, accompanied by an increase in false positives. (Green box: Ground truth of colorectal polyp in the image, Red box: Model-generated bounding box of colorectal polyp, Number: Predicted confidence score by the model).

The polyp localization model demonstrated an F_1_-score of localization task of 0.857 on normal RGB images and 0.883 on modified 1.0R-1.0G-0.8B images. False positive results of the model were 196 and 376 from 5139 sample boxes on original color images and the modified 1.0R-1.0G-0.8B images respectively (Table 3) (Fig. 2, Fig. 3, Fig. 4).

#### The performance of the polyp localization model on external validation

The polyp localization model demonstrated an F_1_-score of 0.535 and 0.556 on original color images and the modified 1.0R-1.0G-0.8B images respectively. False positive results of the model were 1944 and 4735 from 22,364 sample boxes on original color images and the modified 1.0R-1.0G-0.8B images respectively (Table 4).

### Discussion

In this study, we compared the performance of the polyp detection and polyp localization models on adjusted RGB images with original color images. RGB adjustment, particularly the reduction of the blue channel, improved the performance of polyp detection by approximately 1 % in mAP and 2–3 % in the F_1_-score for polyp localization, albeit with an increase in false positive results. While RGB adjustment enhanced the mAP for adenomatous polyp detection, it showed minimal improvement for hyperplastic polyp detection.

Decreasing the blue color channel proved effective in improving polyp detection, likely due to the common occurrence of pronounced noise in the blue channel. The heightened noise in the blue channel also has an impact on the other channels as well, owing to demosaicing [22]. Reducing the blue channel helped minimize noise impact, allowing the model to extract more features for polyp detection.

The increase in false positives in polyp localization following RGB adjustment may be attributed to the model extracting more features. While this enhances sensitivity, it also raises the likelihood of false positives. As discussed in Section 1.2 of the Method validating section, RGB adjustments improved the detection of adenomatous polyps more significantly than hyperplastic polyps, suggesting that different polyp types may respond differently to color adjustments.

**Fig. 5:**
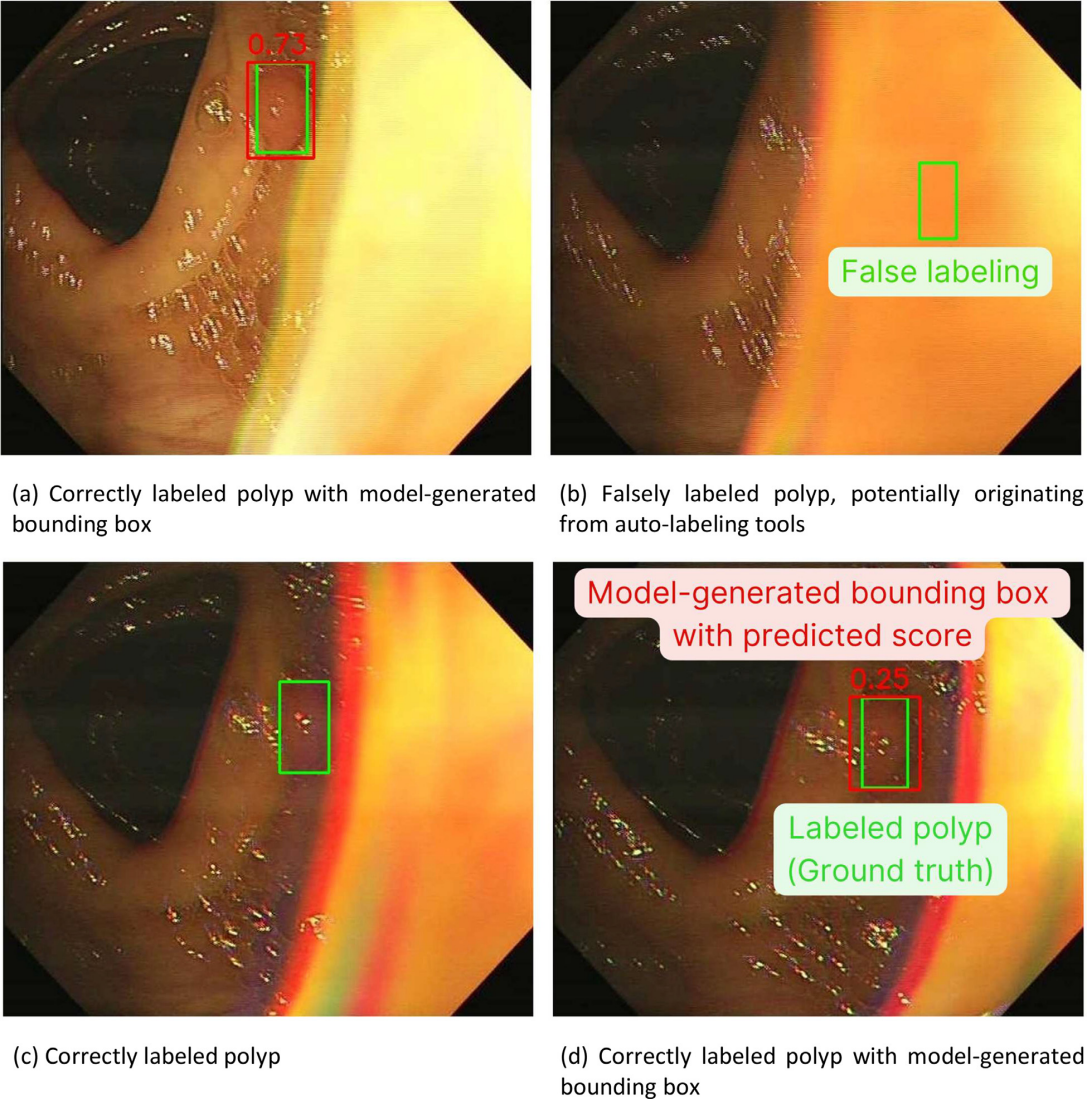
False labeling of the external dataset, potentially originating from auto-labeling tools.

The low performance of the polyp localization model on the external test set, with an F_1_-score of 0.535 for original color images and 0.556 for modified 1.0R-1.0G-0.8B images, may be due to false labeling from auto-labeling tools used in the LDPolypVideo-Benchmark [14]. In some pictures in the dataset, polyps were obscured behind the colorectal wall. However, the bounding boxes were located on the colorectal wall (Fig. 5). In addition, localization errors also occurred in images featuring inconspicuous polyp protrusion, excessive noise or motion artifacts, and small pixel sizes of polyps.

The result on normal RGB, which achieved the mAP of 0.761, was in line with the previous work on this dataset, which received a mAP of 0.735 with RefineDet [12]. The improved performance of polyp detection model mAP after preprocessing was also consistent with the findings of Chen-Ming Hsu et al. and Zhiqin Qian et al. even though the preprocessing was different. This highlighted the impact of image preprocessing in the performance of polyp detection and localization models.

Our study is one of the very first studies that determine the effect of RGB adjustment on the performance of polyp detection model and polyp localization model. This study also used the two large public datasets to train, test, and external validate the models. The improved performance of the models from RGB adjustment provides valuable insights into optimizing image preprocessing for polyp detection and localization. However, the study is limited by the variability in performance across external validation datasets and polyp type.

Further work should focus on validating the generalizability of RGB adjustments in diverse clinical settings, as well as integrating multiple preprocessing technique to a tailored polyp detection/localization model to maximize the capability of the artificial intelligence in polyp detection and localization. Increasing the performance of the model on adenomatous polyps is also needed to study. Additionally, the real-world applications should be tested since the computational burden and processing time may limit the practicality of this approach. To address the increased false positives in polyp localization after RGB adjustment, further analysis is needed to identify specific causes and mitigate this effect. This could involve conducting feature and error analysis, refining the model, exploring ensemble methods, and implementing more robust cross-validation and external validation techniques to ensure the model's performance is consistent and generalizable. Exploring ensemble methods and hybrid preprocessing techniques, such as combining RGB adjustment with HSV adjustment or noise reduction, could also enhance feature extraction and improve overall model performance [23] Table 1.

**Table 1:** The performance (mAP50) of YOLOv8s model on different amount of training set of normal RGB images.

Training set proportion (%)	mAP_50_
100	0.747
60	0.750
40	0.734
20	0.713
10	0.678

**Table 2:** Performance of polyp detection (classification and localization) model.

		Precision	Sensitivity	F_1_	mAP_50_
1.0R-1.0G-1.0B images	All	0.689	0.702	0.695	0.761
	Adenomatous	0.522	0.670	0.587	0.671
	Hyperplastic	0.855	0.735	0.790	0.851
1.1R-1.1G-1.0B images	All	0.722	0.683	0.702	0.767
	Adenomatous	0.585	0.647	0.614	0.680
	Hyperplastic	0.859	0.719	0.783	0.855
1.0R-1.0G-0.8B images	All	0.727	0.714	0.720	0.777
	Adenomatous	0.639	0.656	0.647	0.700
	Hyperplastic	0.814	0.772	0.792	0.854

**Table 3:** Performance of polyp localization model on internal validation and internal test set.

	Precision	Sensitivity	F_1_	False positive	Sample (boxes)
Internal validation					
1.0R-1.0G-1.0B images	0.969	0.920	0.944	137	4584
1.0R-1.0G-0.8B images	0.936	0.966	0.951	304	4584
Internal test set					
1.0R-1.0G-1.0B images	0.953	0.778	0.857	196	5139
1.0R-1.0G-0.8B images	0.921	0.849	0.883	376	5139

**Table 4:** Performance of polyp localization model on external test set.

	Precision	Sensitivity	F_1_	False positive	Sample (boxes)
External test set					
1.0R-1.0G-1.0B images	0.820	0.397	0.535	1944	22,364
1.0R-1.0G-0.8B images	0.688	0.467	0.556	4735	22,364

## Limitations

Increased False Positives: The RGB color adjustment led to an increase in false positives in polyp localization, which could affect the precision of the model.

Variable Performance Across Datasets: The performance of the model varied across different datasets, particularly in external validation, indicating that the model may not generalize well to all types of colonoscopic images.

Dataset Labeling Errors: Some labeling errors in the external validation dataset, such as polyps obscured behind the colorectal wall, may have impacted the model's performance.

Computational Burden: The process of RGB adjustment and model training can be computationally intensive, which may limit its practicality in real-time applications.

Need for Further Validation: The improvements observed need to be validated across diverse clinical settings and with different datasets to ensure the robustness and generalizability of the findings.

## Conclusion

RGB color adjustment can enhance CNN model performance for colorectal polyp detection and localization. Although promising, variable performance across external datasets underscores the need for further research to validate these findings and optimize preprocessing techniques for broader clinical applications.

## Ethics statements

In accordance with the Declaration of Helsinki and the International Conference on Harmonization in Good Clinical Practice, this study was approved by the institutional review board of King Mongkut's Institute of Technology Ladkrabang, Bangkok, Thailand on June 9, 2023 (EC-KMITL_66_068).

## Supplementary material *and/or* additional information

None.

## CRediT authorship contribution statement

**Jirakorn Jamrasnarodom:** Conceptualization, Methodology, Validation, Formal analysis, Investigation, Resources, Data curation, Writing – original draft, Visualization, Project administration, Writing – review & editing. **Pharuj Rajborirug:** Methodology, Software, Data curation, Visualization, Writing – review & editing. **Pises Pisespongsa:** Methodology, Validation, Supervision, Writing – review & editing. **Kitsuchart Pasupa:** Conceptualization, Methodology, Validation, Formal analysis, Supervision, Writing – review & editing.

## Declaration of competing interest

The authors declare that they have no known competing financial interests or personal relationships that could have appeared to influence the work reported in this paper.

## Data Availability

We used public dataset in this study.
